# The unusual reproductive system of head and body lice (Pediculus humanus)

**DOI:** 10.1111/mve.12287

**Published:** 2017-12-20

**Authors:** A. G. DE LA FILIA, S. ANDREWES, J. M. CLARK, L. ROSS

**Affiliations:** ^1^ School of Biological Sciences, Institute of Evolutionary Biology University of Edinburgh Edinburgh U.K.; ^2^ Departnent of Veterinary and Animal Sciences University of Massachusetts Amherst Amherst MA U.S.A.

**Keywords:** Pediculus humanus, human louse, paternal genome elimination, pseudohaplodiplody, resistance evolution

## Abstract

Insect reproduction is extremely variable, but the implications of alternative genetic systems are often overlooked in studies on the evolution of insecticide resistance. Both ecotypes of Pediculus humanus (Phthiraptera: Pediculidae), the human head and body lice, are human ectoparasites, the control of which is challenged by the recent spread of resistance alleles. The present study conclusively establishes for the first time that both head and body lice reproduce through paternal genome elimination (PGE), an unusual genetic system in which males transmit only their maternally derived chromosomes. Here, we investigate inheritance patterns of parental genomes using a genotyping approach across families of both ecotypes and show that heterozygous males exclusively or preferentially pass on one allele only, whereas females transmit both in a Mendelian fashion. We do however observe occasional transmission of paternal chromosomes through males, representing the first known case of PGE in which whole‐genome meiotic drive is incomplete. Finally, we discuss the potential implications of this finding for the evolution of resistance and invite the development of new theoretical models of how this knowledge might contribute to increasing the success of pediculicide‐based management schemes.

## Introduction

One of the most striking features of insects is the extraordinary diversity of their reproduction, which is unparalleled in any other animal group. This is illustrated by the wide heterogeneity of reproductive and genetic systems found across insect taxa that differ from the canonical diplodiploidy prevalent in metazoans (Normark, 2003). The most well‐known example of these alternative genetic systems is arguably arrhenotoky (i.e. haplodiploidy *sensu stricto*, whereby males develop from unfertilized eggs). However, many other more complex and bizarre non‐diplodiploid systems have been described. Many of these are common in economically important insects: for instance, parthenogenesis (female reproduction without fertilization) is disproportionately abundant in pest species, including representatives of groups such as mites, aphids and scale insects, compared with non‐pest relatives (Hoffmann *et al*., 2008; Ross *et al*., 2013). Telling signs of alternative genetic systems are non‐Mendelian inheritance patterns of traits or genetic markers, which are often discovered fortuitously in certain species but are rarely explored further despite their potential implications for key aspects of insect management, such as the evolution of virulence and insecticide resistance.

One of these species is the human louse, *Pediculus humanus*, a blood‐sucking ectoparasite that occurs worldwide and causes infestations with serious medical, economic and social consequences. Human lice are divided into two ecotypes: the head louse (*Pediculus humanus capitis*) and the body louse (*Pediculus humanus humanus*) (Durden & Musser, 1994), which differ in their ecology and clinical importance. Whereas body lice feed on human skin and lay eggs on clothes, head lice live and feed on the human scalp only. Epidemiologically, head louse infestations are more common and mostly affect children, regardless of economic status or geographic region (Clark *et al*., 2013). By contrast, body louse infestations are associated with lower socioeconomic conditions and pose a more serious health threat because the body louse is a vector of epidemic pathogenic bacteria, including *Bartonella quintana* (trench fever), *Borrelia recurrentis* (relapsing fever) and *Rickettsia prowazekii* (epidemic typhus) (Raoult & Roux, 1999).

Control of human lice generally involves a combination of manual removal techniques and the use of diverse chemicals often referred to as pediculicides. However, many of the most widely used pediculicides have become ineffective as a result of the spread of resistant strains [see Durand *et al*. (2012) and references therein] and, as many pediculicides share common chemistry and targets (Clark *et al*., 2013), further spread of resistance is likely. To reduce this risk, it is important to unravel the molecular and metabolic mechanisms involved in pediculicide resistance (Oakeshott *et al*., 2003), which have been addressed by several studies in recent years (Yoon *et al*., 2008; Kwon *et al*., 2014). However, current understanding of how resistance evolves and spreads through populations is very limited because key factors such as population structure, gene flow, reproductive genetics, life history and mating system remain insufficiently explored. Better understanding of these factors and their roles in the evolution of pesticide resistance will support the development of successful novel treatment strategies and management programmes aimed at preventing the spread of resistance genotypes.

Until recently, it was assumed that inheritance of traits such as pesticide resistance in lice would follow the classic laws of Mendelian genetics. However, an unexpected finding in the body louse suggested that whereas allele transmission in females followed Mendelian expectations, it was non‐Mendelian in males: heterozygous male parents systematically passed on one of their two alleles to their offspring (McMeniman & Barker, 2005). Moreover, the transmitted allele was of maternal origin in all cases and the paternally derived alternative allele was absent from the offspring. This mode of inheritance is consistent with paternal genome elimination (PGE), a type of haplodiploid reproduction found across several arthropod orders in which males do not transmit paternally inherited alleles to their offspring (Normark, 2003). It is surprising that the possible presence of PGE in *P. humanus* has not been considered in the context of louse control because it may have implications for the evolution of pesticide resistance. Theoretical approaches have shown that haplodiploidy can accelerate the invasion of resistant alleles under certain circumstances (Crozier, 1985; Caprio & Hoy, 1995; Denholm *et al*., 1998; Carrière, 2003), and PGE has been invoked to explain the rapid spread of insecticide resistance in New Caledonian populations of the coffee berry borer beetle *Hypothenemus hampei* (Brun *et al*., 1995). Furthermore, PGE is likely to elicit sex‐specific responses and selection pressures that can further affect the way resistance genotypes spread through populations (Carrière, 2003).

Although the study by McMeniman & Barker (2005) is suggestive of the presence of PGE in *P. humanus*, it requires further confirmation. They show that a proportion of heterozygous males transmit both alleles in a Mendelian fashion, which would mean that PGE was polymorphic in the study population (McMeniman & Barker, 2005). This finding is unlike any form of PGE described so far, which has always been found to be complete. Further, McMeniman & Barker (2005) used only three markers in their study, which falls short of covering the whole genome and does not allow determination of whether drive is complete or restricted to some chromosomes. Moreover, the Culpepper strain (Culpepper, 1944) used by McMeniman & Barker (2005) in their experiment might not be representative of natural populations as it has evolved under laboratory conditions since 1945 and has adapted to rabbit blood, rather than human. It is therefore possible that a drive factor emerged in this strain independently of natural body louse populations, which were not sampled. Finally, the study by McMeniman & Barker (2005) included only body lice and no data on inheritance in head lice have been published since. Here, we study patterns of allele inheritance in both head and body louse families derived from recently collected natural populations reared on human blood.

In order to determine whether males show complete genome‐wide meiotic drive consistent with PGE, we used a two‐generation experimental crossing design and a panel of multiple polymorphic microsatellite markers. Transmission patterns were determined by genotyping both parents and their offspring to determine whether both alleles at a given heterozygous parental locus are present at a 50 : 50 ratio in the offspring (Mendelian transmission) or whether only one allele is transmitted by male parents (PGE). The current study provides the first reported evidence of PGE in the head louse and confirms its occurrence in body lice, albeit with no consistent evidence of a PGE polymorphism between males. We do, however, observe occasional leakage of paternal alleles, especially in body lice. Finally, we also suggest subsequent research directions aimed at increasing current understanding of how PGE operates in lice, particularly whether it affects gene expression patterns in males, and discuss the implications of this unusual genetic system for the evolution of parasitic lice in general and, most specifically, the evolution of pediculicide resistance.

## Materials and methods

### Experimental design

A series of intraspecific crosses were set up using individuals from the head louse strain SF‐HL and the body louse strain Frisco‐BL. The SF‐HL colony was established in 2002 from head lice collected from ∼ 20 infested children in Plantation, Miami and Homestead (FL, U.S.A.). Approximately 50 males and 50 females were used to temporarily establish a colony on human volunteers (Takano‐Lee *et al*., 2003). Fertile eggs from Homestead were added to the colony at least three times between 2002 and 2006. Approximately 30–50 eggs were introduced each time. The sex ratio of the eggs was assumed to be ∼ 50 : 50. The colony was placed on an *in vitro* rearing system in 2006 (Yoon *et al*., 2006). The Frisco‐BL colony of human body lice was originally collected from nine homeless individuals in San Francisco (CA, U.S.A.) by Dr Jane Koehler (University of California San Francisco Medical Center, San Francisco, CA, U.S.A.) in December 2008. Both colonies have been maintained by the Clark Laboratory at the University of Massachusetts‐Amherst on human blood using the same *in vitro* rearing system (Yoon *et al*., 2006) under environmental conditions of 30 °C, 70% relative humidity and an LD 16 : 8 h photoperiod in rearing chambers (University of Massachusetts‐Amherst Institutional Review Board approval no. E1404/001‐002).

Parental generations (F_0_) were established by random selection of pairs of sexually immature third instar lice from each colony. These pairs were transferred to individual cages. Lice were sexed after reaching reproductive maturity using the approach first described by Meinking (1999) and cages were checked for same‐sex pairs. In these cases, a pair of male‐only and female‐only cages was selected at random and a randomly chosen individual was swapped between cages. After this point, all cages were screened daily to check for oviposition or the death of parents. Males were removed and stored in 100% ethanol at 4 °C after 7 days or immediately after their death. Females were allowed to lay eggs for 2 weeks and were then removed and stored in 100% ethanol at 4 °C. Offspring (F_1_) of all crosses were raised until early third instar stage and then transferred to ethanol. In total, F_1_ broods for 26 head and 13 body louse families were obtained.

### DNA extraction and polymerase chain reaction

Total genomic DNA from parents and body louse F_1_ individuals was extracted with a DNeasy Blood and Tissue Extraction Kit (Qiagen Benelux BV, Venlo, the Netherlands). DNA from head louse F_1_ individuals was extracted with a prepGEM Insect Kit (ZyGEM NZ Ltd, Hamilton, New Zealand) in a 20‐µL reaction volume. A panel of three multiplexes (MX1, MX2 and MX4) from Ascunce *et al*. (2013) containing 12 microsatellite loci in total (T8_1, M3_10, M3_19, M2_2, T2_6, M2_19, M2_13, M2_3, T9_6, T2_7, T4_5 and T1_4) was used for polymerase chain reaction (PCR) amplification. The PCR reactions for each of the three multiplexes were performed using the Type‐it Microsatellite PCR Kit (Qiagen Benelux BV) in a 15‐µL reaction volume. Primer sequences and reaction mixes were as described in supplementary Tables S1–3 in Ascunce *et al*. (2013). The PCR reactions were performed under the following conditions: initial denaturation at 95 °C for 5 min; 35 cycles of denaturation at 94 °C for 30 s; annealing at 52 °C for 45 s; extension at 72 °C for 45 s, and a final extension step at 72 °C for 40 min. One microlitre of PCR product for each sample and multiplex was sent to Edinburgh Genomics (University of Edinburgh) for genotyping on the ABI 3730 DNA Analyzer system (ThermoFisher Scientific, Inc., Waltham, MA, U.S.A.).

### Primer mapping

To reveal the extent of the genome coverage of the microsatellite panel in use, all loci were mapped against the most recent publicly available louse genome assembly. All forward and reverse primer sequences were blasted against the U.S. Department of Agriculture strain genomic assembly (PhumU2) using the blast tool in VectorBase (National Institute of Allergy and Infectious Diseases, National Institutes of Health, Bethesda, MD, U.S.A.) with default settings.

### Microsatellite scoring and data analysis

Upon reception of raw trace files, microsatellite alleles were scored using the Microsatellite Plugin implemented in geneious Version 8.1.3 (Biomatters Ltd, Auckland, New Zealand). Estimates of observed (H_O_) and expected (H_E_) heterozygosity, number of alleles and inbreeding coefficient F_IS_ (Weir & Cockerham, 1984) per locus for F_0_ populations were obtained using the online version of genepop Version 4.2 (Raymond & Rousset, 1995; Rousset, 2008) with default parameters. For each family and locus, paternal and maternal allele transmission ratios were calculated as the number of occurrences of one of the two alleles in the F_1_ offspring divided by the total number of F_1_ genotypes. Because of the clear expectation of allele transmission following McMeniman & Barker (2005) and other PGE species, these parental ratios were defined in different ways to represent these different sex‐specific transmission patterns. For paternal transmission ratios, the allele used in this calculation was that with higher representation in the offspring genotypes. For maternal transmission ratios, one of the two alleles was chosen at random. Likewise, when both parents were heterozygous for the same alleles at a given locus, parental allele counts were assigned under the assumption that the driving allele present in all offspring was paternally derived. Exact binomial tests to detect significant deviations from Mendelian expectations in each locus were performed in R Version 3.2.4 (R Foundation for Statistical Computing, Vienna, Austria). To correct for multiple testing, a reduced significance level of α = 0.01 is considered in addition to the conventional level of α = 0.05.

## Results

### Informative parents and microsatellite panel

In order to determine patterns of allele transmission, the F_1_ offspring of F_0_ parents with at least one heterozygous locus were genotyped because parents that are homozygous for all loci are not informative. Multi‐locus heterozygosity of parental populations was higher in head louse F_0_ (H_O_ = 0.351) than in body louse F_0_ (H_O_ = 0.256) despite higher allelic richness in the latter, as a result of the elevated inbreeding coefficient in the body louse population (F_IS_ > 0.2) (Table 1). At least one heterozygous marker was found in all 26 head louse and 11 body louse fathers. Likewise, 24 head louse and all 13 body louse mothers were heterozygous for at least one locus. This allowed for the determination of both paternal and maternal allele transmission patterns in almost all families (Table S1, online).

**Table 1:** Multi‐locus descriptive statistics of head and body louse F_0_ parental populations.

Species	Families	F_1_/family	Loci	Allele/locus	H_O_	H_E_	F_IS_	H_O_ ♂	H_O_ ♀
Head louse	26	8–12	11 (10)	2.55 ± 0.32	0.341 ± 0.065	0.366 ± 0.071	0.021 ± 0.044	0.315 ± 0.064	0.367 ± 0.069
Body louse	13	7–22	9 (9)	3.00 ± 0.21	0.256 ± 0.056	0.336 ± 0.051	0.262 ± 0.101	0.239 ± 0.064	0.274 ± 0.067

Families, number of F_0_ parental pairs establishing F_1_ broods.

Loci, number of reliable loci included in the analysis (informative; i.e. polymorphic loci in parentheses).

F_1_/family, range of number of individuals per family genotyped for each ecotype.

Allele/locus, mean ± standard error (SE) number of alleles per marker.

H_O_, mean ± SE observed heterozygosity across all loci.

H_E_, mean ± SE expected heterozygosity across all loci.

F_IS_, mean ± SE F_IS_ across all loci (following Weir & Cockerham, 1984).

H_O_ ♂ and H_O_ ♀, mean ± SE observed heterozygosity across all loci for F_0_ fathers and F_0_ mothers.

All F_0_ and F_1_ individuals were genotyped using the 12‐locus microsatellite panel, but not all markers could be included in the analysis (Tables S2 and S3, online). T9_6 was monomorphic in head lice, whereas T9_6 and T1_4 failed to amplify in most body louse individuals and were excluded in this ecotype, but all remaining loci were polymorphic and amplified consistently in most families. It was further decided that the T8_1 locus should be excluded in both ecotypes as a result of genotype inconsistencies. Therefore, from the initial 12‐locus microsatellite panel, 10 and nine reliable informative loci were used to estimate allele transmission patterns in head and body louse families, respectively.

To assess the genome coverage of the microsatellite panel, all primer sequences were blasted to the *P. humanus* genome assembly to determine whether they were located in different genomic regions. Each of the markers was found to map to a distinct scaffold in the genome assembly (Table 2). Although the genome assembly does not allow for the exact determination of which chromosomes are targeted by the markers used herein, the present authors are confident that the panel offers sufficient coverage for a genomewide study of transmission patterns. By contrast, very limited genome coverage of the three markers used in McMeniman & Barker (2005) was found because two of them map to the same scaffold and the location of the third is unclear.

**Table 2:** Genome location of markers developed by Ascunce et al. (2013) (used in this study) and Leo et al. (2002) [used in McMeniman & Barker (2005)].

Panel	Locus	Scaffold	*E*‐value
Ascunce *et al*. (2013)	M3_10	DS235157	0.002
	M3_19	DS235833	0.002
	M2_2	DS235048	0.0005
	T2_6	DS235090	0.0002
	M2_19	DS235875	0.0005
	M2_13	DS235785	0.002
	M2_3	DS235111	0.0005
	T9_6	DS235882	< 0.0001
	T2_7	DS235100	0.002
	T4_5	DS235283	0.0002
	T1_4	DS235023	0.002
Leo *et al*. (2002)	ML_8	DS235886	< 0.0001
	ML_9	DS235886	0.002
	ML_10	DS235042*	0.023
		DS235005†	0.98

All forward and reverse primers for each locus mapped to the same scaffold; the highest *E*‐value for each of the pairs is shown, except for ML_10 (*forward; †reverse).

### Allele transmission patterns in males and females

**Figure 1:**
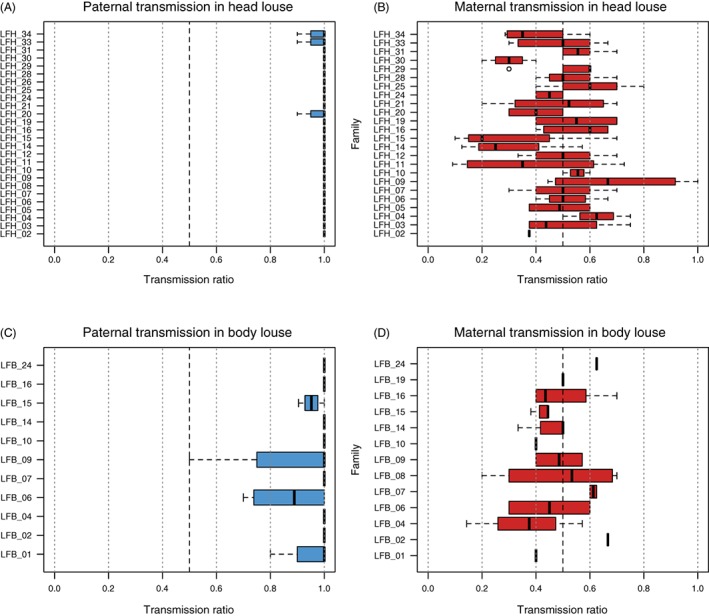
Allele transmission ratios across all loci for head and body louse (A, C) males and (B, D) females. When both alleles are equally represented in F_1_ offspring, the transmission ratio is 0.5 (complete Mendelian transmission). A transmission ratio of 1 indicates complete drive of one of the parental alleles. [Colour figure can be viewed at http://wileyonlinelibrary.com].

For most families and loci in both ecotypes, heterozygous head and body louse males did not transmit alleles in a Mendelian fashion, but consistently passed on only one allele to the F_1_. By contrast, females transmit both alleles to their offspring (Fig. 1, Table S1). These patterns are consistent with PGE: females are normally diploid and exhibit Mendelian transmission, whereas males show whole‐genome drive and transmit only the maternally inherited allele at each locus.

**Figure 2:**
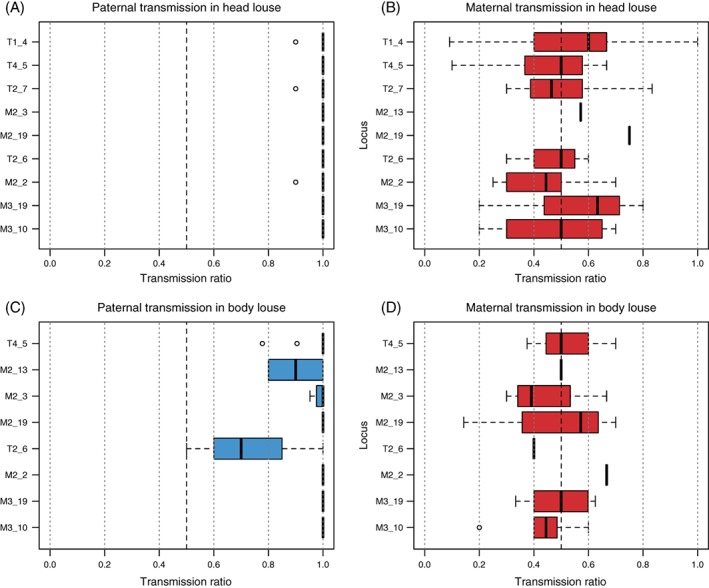
Paternal and maternal allele transmission ratios in all (A, B) head and (C, D) body louse families grouped by loci. [Colour figure can be viewed at http://wileyonlinelibrary.com].

However, despite clear preferential transmission of one of the two alleles at each locus, head and body louse males sporadically also transmitted alternative (i.e. paternally inherited) alleles. Occasional paternal transmission of alternative alleles was observed across most markers, except for M2_19, M3_19 and M3_10 (Fig. 2). Escapes were rare in head louse males: three males (LFH_20, LFH_33 and LFH_34) passed on an alternative allele once at a single different locus (T1_4, M2_2 and T2_7, respectively). The other 23 head louse males showed complete PGE at all heterozygous loci. Overall, 64 of 71 head louse paternal transmission ratios deviated significantly from the Mendelian expectation of equal transmission at a significance threshold of 0.01 (all 71 at α = 0.05), compared with one of 81 ratios in head louse females.

In body louse families, incomplete PGE occurrences were more frequent. Four of the 11 informative males also transmitted the alternative allele at least once (LBH_01 and LBH_09 at one locus, LBH_06 and LBH_15 at two loci). With a significance threshold of 0.01, 19 of 28 ratios deviated from Mendelian transmission (25 of 28 at α = 0.05). In body louse females, none of the 29 transmission ratios deviated from Mendelian expectations.

The present study did not find a consistent pattern of incomplete PGE instances across families and loci. To exclude genotyping error for these unexpected paternal escapes, both parents and offspring were re‐genotyped and additional offspring were genotyped when available. We are therefore confident that the current findings represent true events of paternal chromosomes escaping germline elimination at low frequencies, particularly in body lice.

## Discussion

The allele transmission patterns described in the present study offer conclusive evidence of a genome‐wide male transmission ratio distortion in both ecotypes of *P. humanus*: males exclusively (or, in some cases, preferentially) transmit only one of their alleles to their offspring. In addition, heterozygous genotypes in males from both ecotypes unambiguously indicate that males are diploid and that both paternally and maternally inherited chromosomes are kept in the soma. Although the two‐generation experimental design used in this study does not explicitly allow for determination of the parental origin of alleles in F_0_ individuals, McMeniman & Barker (2005) already demonstrated that driving alleles were always maternally inherited in body louse males. All these findings are consistent with germline PGE, a pseudohaplodiploid genetic system in which males develop from fertilized eggs and are diploid, but eliminate chromosomes of paternal origin from their germline. This type of reproduction is also found in several other insect taxa such as mealybugs, the coffee borer beetle, two dipteran clades and book lice (Burt & Trivers, 2006; Gardner & Ross, 2014; de la Filia *et al*., 2015; Hodson *et al*., 2017).

All males in the present study exhibited whole‐genome transmission ratio distortion with sporadic, inconsistent leakages of non‐driving alleles in some individuals. Interestingly, the current data reveal that leakages are more frequent in body than in head lice, although the power to detect these occurrences was greater in the latter because twice as many head louse families were screened and they showed higher levels of heterozygosity. However, the study found no evidence of a female PGE‐inducing genetic polymorphism as suggested by McMeniman & Barker (2005). In their model, a codominant maternally transmitted genetic element is responsible for elimination of paternal alleles in male offspring so that females that are heterozygous for this element produce PGE sons that pass on only maternal alleles and non‐PGE sons that transmit parental alleles in a Mendelian fashion. However, the mapping of markers to the louse genome revealed that McMeniman & Barker (2005) appear to have targeted a single chromosome only. Therefore, an alternative interpretation of these earlier results that is consistent with the sporadic leakage of paternal alleles observed in the current study would be a germline PGE mechanism in which discrimination between maternal and paternal chromosomes during spermatogenesis is not infallible. In germline PGE, males are somatically diploid and elimination of paternal chromosomes is achieved via non‐random assortment of chromosomes during meiosis so that only nuclei containing maternal chromosomes develop into viable sperm (Burt & Trivers, 2006). Whole‐genome meiotic drive of maternal chromosomes in germline PGE taxa has been most extensively described in sciarid flies (Esteban *et al*., 1997; Goday & Esteban, 2001) and mealybugs (Bongiorni *et al*., 2004, 2009). Allele transmission patterns in louse males reveal that paternal chromosomes are similarly excluded from active spermatocytes, but are occasionally able to escape elimination by migrating with other maternal chromosomes in lieu of their homologues, particularly in body lice. Achiasmatic male meiosis, which is an imperative requisite for PGE as it prevents mixing of paternal and maternal alleles, has been documented in lice (Tombesi & Papeschi, 1993; Tombesi *et al*., 1999; Bressa *et al*., 2015). As recombination between maternal and paternal homologues cannot account for transmission of paternal alleles, the detected leakage would encompass entire paternal chromosomes. Therefore, the apparent non‐PGE body louse males found by McMeniman & Barker (2005) are more likely to be males exhibiting biparental transmission for the chromosome targeted by their marker panel only, whereas transmission of other chromosomes consistent with PGE (or additional occurrences of paternal leakages) would have passed undetected.

Head and most particularly body lice are the first species for which incomplete (albeit not polymorphic) PGE has been explicitly reported. The discrimination mechanism by which paternal and maternal louse chromosomes are differentially tagged is unknown. In other PGE taxa, maternal and paternal chromosomes differ in patterns of DNA methylation (Bongiorni *et al*., 1999, 2009) and histone modifications (Goday & Ruiz, 2002; Greciano & Goday, 2006; Khosla *et al*., 2006; Escribá *et al*., 2011; Prantera & Bongiorni, 2012), which may mediate discrimination between homologues during spermatogenesis. In lice, inaccuracies of the parent‐of‐origin discrimination mechanism, whichever its nature, could result in the occasional migration of paternal chromosomes with the remaining maternal chromosomes.

Although at this stage the issue of how these leakages occur is subject only to speculation, a likely PGE mechanism in which only nuclei containing maternal chromosomes develop into viable sperm (bar accidental leakage of paternal homologues) can be proposed based on previous cytogenetic work in lice. Louse spermatogenesis is highly complex: achiasmatic meiosis is followed by three or four mitotic divisions to yield a 32/64‐cell cyst that undergoes a final and unequal mitosis in which most cytoplasmic material is excluded from half the cells, which degenerate into pyknotic nuclei (Hindle & Pontecorvo, 1942; Bressa *et al*., 2015) similar to those seen in mealybug spermatogenesis (Bongiorni *et al*., 2004, 2009). The present authors agree with McMeniman & Barker (2005) that non‐random assortment of chromosomes is likely to occur in the last, unequal division, after which only the spermatids carrying maternal chromosomes develop into viable spermatozoa. This implies an inverted meiotic sequence in which the first division is equational rather than reductional, with sister chromatids separating before segregation of homologous chromosomes, as found in other PGE taxa such as mealybugs (Viera *et al*., 2008). It is possible that inverted meiosis in louse males has been historically overlooked in cytogenetic studies as a result of the lack of heteromorphic bivalents and the tight association and highly condensed nature of louse chromosomes, which are holocentric [i.e. they lack a localized centromere; see Bressa *et al*. (2015) and references therein]. Recently, Bressa *et al*. (2015) reported that sister chromatid separation indeed occurs in the first division, but conclusive evidence has yet to be presented.

PGE may have important implications for the transmission of pesticide resistance, which must be parent‐of‐origin‐dependent in males. Resistant males are unable to pass on the trait to their offspring when it is paternally derived and hence resistance will be lost through the paternal line even if it is under strong positive selection. By contrast, males that inherited the resistance trait from their mothers will transmit it to all their offspring, rather than half as occurs in Mendelian inheritance. These characteristic PGE inheritance patterns complicate predictions of resistance invasion without models that explicitly consider sex‐specific differences on allelic transmission. In addition, PGE also reduces effective population sizes (Wright, 1933), although this effect may be small when sex ratios are female‐biased (Hedrick & Parker, 1997), as is often the case in louse populations (Perotti *et al*., 2004).

Another way in which PGE can affect the evolution of resistance is through its potential effect on patterns of gene expression. Taxa in which PGE occurs vary in the degree of paternal genome expression in males, which can affect response to insecticides and have an impact on rates of resistance evolution. In many PGE groups, paternal chromosomes are lost (haploid soma PGE) or transcriptionally inactive (functionally haploid PGE) (Normark, 2003). One immediate consequence of these two forms of PGE is that maternally inherited recessive alleles are directly exposed to selection in males, as under arrhenotoky. Therefore, the evolution of insecticide resistance is faster in arrhenotokous (Crozier, 1985; Havron *et al*., 1987; Caprio & Hoy, 1995; Denholm *et al*., 1998) and functionally haploid PGE species (Brun *et al*., 1995) than in diplodiploids [but not always; see Carrière (2003)]. However, males in other PGE taxa are diploid and may express both alleles regardless of parental origin (diploid soma PGE) (Gardner & Ross, 2014).

Because of this variation in gene expression patterns in PGE systems, it is important to precisely determine the degree of paternal genome expression in louse males. Although heterozygous males show that paternal chromosomes are retained, it is still possible that these are transcriptionally inert. In functionally haploid PGE taxa that remain somatically diploid, inactive paternal chromosomes appear as highly compacted dots (Brown & Nur, 1972; Brun *et al*., 1995; Hodson *et al*., 2017). To the present authors' knowledge, this conspicuous chromosomal behaviour has never been described in human lice, which suggests that PGE is of the diploid soma form and paternal chromosomes are hence transcriptionally active. Phenotypic assays in hybrid individuals are other indicators of paternal chromosome expression in PGE males because they are expected to show the same traits as males from the maternal species if paternal chromosomes are inactivated. Body size and tibia length measurements in hybrids have been reported to be intermediate between head and body lice (Busvine, 1978), but this study did not discriminate between male and female offspring.

If paternal chromosomes are expressed in human louse males, the aforementioned theoretical models on evolution resistance in haplodiploids cannot be applied because they do not consider diploid expression in PGE species with arrhenotokous‐like inheritance. Therefore, new theory must be developed to predict how whole‐genome meiotic drive in males with diploid gene expression will affect resistance evolution.

How PGE evolved in the human louse remains an open question. Although *P. humanus* is the only anopluran (i.e. sucking louse) in which the occurrence of PGE has been explicitly demonstrated, the same modified spermatogenesis has been reported in other parasitic louse species. These include another anopluran, the pig louse *Haematopinus suis* (Phthiraptera: Haematopinidae) (Bayreuther, 1955; Tombesi & Papeschi, 1993), and members of two suborders of the paraphyletic group Mallophaga (i.e. chewing lice): Amblycera [the guinea pig louse *Gyropus ovalis* (Phthiraptera: Gyropidae) and the chicken body louse *Menacanthus stramineus* (Phthiraptera: Menoponidae)] (Scholl, 1955; Tombesi & Papeschi, 1993) and Ischnocera [two species of *Bovicola* (Phthiraptera: Trichodectidae), the goat louse] (Tombesi *et al*., 1999). More tellingly, empirical evidence of PGE in a close relative of parasitic lice, the book louse *Liposcelis* sp. (Psocoptera: Liposcelididae), has been recently provided (Hodson *et al*., 2017). In this species, PGE is of the functionally haploid type, with males retaining condensed paternal chromosomes. Although the phylogenetic relationships between and within Psocoptera (book lice) and Phthiraptera are not yet fully resolved and this division has been called into question (Yoshizawa & Johnson, 2010; Li *et al*., 2015), there is consensus that all lice form a monophyletic group and it is therefore possible that PGE is common to all of them. Formal investigation of transmission patterns and somatic heterochromatinization in these or other parasitic louse species would be necessary to corroborate this hypothesis.

Several authors have suggested that PGE may have evolved through attempts by endosymbionts to manipulate their host's reproduction (Normark, 2004; Kuijper & Pen, 2010; Ross *et al*., 2012). The rationale for this is that maternally transmitted endosymbionts benefit from a female‐biased sex ratio and that the elimination of paternally derived chromosomes in males may be a way of killing male offspring. Lice harbour several maternally inherited endosymbiotic bacteria including both obligate nutritional mutualists as well as bacteria known to manipulate host reproduction in their own favour, such as *Wolbachia*. Hence, could PGE in lice be induced by endosymbionts? Probably not: human louse males remain diploid throughout development and only eliminate their paternally derived genome during spermatogenesis, which is unlikely to induce male‐specific mortality and is therefore not in the interest of the endosymbionts.

The present study demonstrates that PGE is present in both *P. humanus* ecotypes and outlines some considerations of the impact of the particular genetic system on the evolution of pediculicide resistance. A more complete understanding of human louse biology is imperative to facilitate the design and application of successful resistance management programmes. Yet asymmetry in gene transmission patterns, sex ratio bias and possible phenotypic consequences of PGE have not been considered thus far. The characterization and compact nature of the *P. humanus* genome enable genome‐wide allele‐specific expression studies to determine the extent to which paternally inherited alleles can confer resistance phenotypes in males. If they can, theoretical models of resistance evolution combining diploid expression and haplodiploid transmission will be needed to maximize the success of resistance control strategies.

## Supporting information


**Table S1.** Paternal and maternal transmission ratios for all families and loci.

**Table S2.** Head louse genotypes.

**Table S3.** Body louse genotypes.
