# Supplementary material for: The unusual reproductive system of head and body lice (Pediculus humanus)
Source: Med Vet Entomol. 2017 Dec 20;32(2):226–34. doi: 10.1111/mve.12287 (PMC5947629; doi:10.1111/mve.12287)
Supplement: Supplementary file 1 — Table S1. Paternal and maternal transmission ratios for all families and loci. [file MVE-32-226-s001.pdf]

Table S1. Paternal and maternal transmission ratios for all families and loci

#F1, number of unambiguous F1 genotypes for each locus.
Pat. A/Pat. B, counts of most common and alternative paternal alleles in F1 genotypes.
Mat. A/Mat. B, counts of both maternal alleles in F1 genotypes chosen randomly.
Pat. TR/Mat. TR, transmission ratio of Pat. A/Mat. A.
P, exact binomial test probability associated between observed and expected counts of Pat. A/Mat. A in F1 under Mendelian expectations.
Significant deviations at the $\alpha = 0.01$ level are highlighted in bold and indicated with two asterisks. Not significant deviations at 0.01 level that are significant at the conventional 0.05 level are indicated with a single asterisk.
NS, not significant at any of these significance level

Family	Locus	# F1	Pat. A	Pat. B	Pat. TR	P		Mat. A	Mat. B	Mat. TR	P	
**Head louse**												
LFH_02	M3_10	7	7	0	1.000	0.016	*	-	-	-	-	
	M2_19	8	8	0	1.000	**0.008**	**	-	-	-	-	
	T2_6	8	-	-	-	-		5	3	0.375	0.727	NS
	T4_5	9	9	0	1.000	**0.004**	**	-	-	-	-	
LFH_03	M3_10	10	10	0	1.000	**0.002**	**	-	-	-	-	
	M3_19	10	10	0	1.000	**0.002**	**	-	-	-	-	
	M2_2	8	8	0	1.000	**0.008**	**	5	3	0.375	0.727	NS
	M2_19	8	-	-	-	-		6	2	0.750	0.289	NS
	T2_7	8	-	-	-	-		5	3	0.375	0.727	NS
	T4_5	8	8	0	1.000	**0.008**	**	4	4	0.500	1.000	NS
LFH_04	M3_10	6	-	-	-	-		3	3	0.500	1.000	NS
	M3_19	7	7	0	1.000	0.016	*	-	-	-	-	
	T2_6	8	8	0	1.000	**0.008**	**	-	-	-	-	
	T1_4	8	-	-	-	-		5	3	0.625	0.727	NS
LFH_05	M3_19	8	-	-	-	-		5	3	0.375	0.727	NS
	T4_5	10	-	-	-	-		6	4	0.600	0.754	NS
	T1_4	10	10	0	1.000	**0.002**	**	-	-	-	-	
LFH_06	M3_19	9	9	0	1.000	**0.004**	**	6	3	0.667	0.508	NS
	M2_3	10	10	0	1.000	**0.002**	**	-	-	-	-	
	T4_5	10	10	0	1.000	**0.002**	**	5	5	0.500	1.000	NS
	T1_4	10	-	-	-	-		6	4	0.400	0.754	NS
LFH_07	M3_19	10	-	-	-	-		7	3	0.700	0.344	NS
	M2_2	10	-	-	-	-		5	5	0.500	1.000	NS
	T2_7	10	10	0	1.000	**0.002**	**	-	-	-	-	
	T4_5	10	10	0	1.000	**0.002**	**	7	3	0.300	0.344	NS
LFH_08	M3_19	8	8	0	1.000	**0.008**	**	-	-	-	-	
	M2_2	8	8	0	1.000	**0.008**	**	-	-	-	-	
LFH_09	M3_19	9	9	0	1.000	**0.004**	**	-	-	-	-	
	M2_2	9	-	-	-	-		5	4	0.444	1.000	NS
	T2_7	6	6	0	1.000	0.031	*	5	1	0.833	0.219	NS
	T4_5	8	-	-	-	-		4	4	0.500	1.000	NS
	T1_4	10	-	-	-	-		10	0	1.000	**0.002**	**
LFH_10	M3_10	10	-	-	-	-		5	5	0.500	1.000	NS
	M3_19	10	10	0	1.000	**0.002**	**	6	4	0.600	0.754	NS
	M2_2	10	10	0	1.000	**0.002**	**	-	-	-	-	
	T4_5	9	9	0	1.000	**0.004**	**	5	4	0.556	1.000	NS
LFH_11	M3_10	10	-	-	-	-		8	2	0.200	0.109	NS
	M3_19	11	11	0	1.000	**0.001**	**	8	3	0.727	0.227	NS
	M2_2	9	9	0	1.000	**0.004**	**	-	-	-	-	
	T4_5	12	12	0	1.000	**<0.001**	**	6	6	0.500	1.000	NS
	T1_4	11	-	-	-	-		10	1	0.091	0.012	*
LFH_12	M3_10	10	-	-	-	-		7	3	0.700	0.344	NS
	M3_19	9	9	0	1.000	**0.004**	**	6	3	0.333	0.508	NS
	T2_7	10	-	-	-	-		6	4	0.600	0.754	NS
	T4_5	10	10	0	1.000	**0.002**	**	6	4	0.400	0.754	NS
	T1_4	10	10	0	1.000	**0.002**	**	5	5	0.500	1.000	NS
LFH_14	M2_2	8	8	0	1.000	**0.008**	**	6	2	0.250	0.289	NS
	M2_19	7	-	-	-	-		4	3	0.571	1.000	NS
	T4_5	8	-	-	-	-		7	1	0.125	0.070	NS
LFH_15	M3_10	10	10	0	1.000	**0.002**	**	7	3	0.700	0.344	NS
	M3_19	10	-	-	-	-		8	2	0.200	0.109	NS
	M2_2	10	10	0	1.000	**0.002**	**	-	-	-	-	
	T4_5	10	10	0	1.000	**0.002**	**	9	1	0.100	0.021	*
	T1_4	10	10	0	1.000	**0.002**	**	-	-	-	-	
LFH_16	M3_10	10	10	0	1.000	**0.002**	**	6	4	0.400	0.754	NS
	T2_6	10	10	0	1.000	**0.002**	**	6	4	0.600	0.754	NS
	T2_7	7	-	-	-	-		4	3	0.429	1.000	NS
	T4_5	9	-	-	-	-		6	3	0.667	0.508	NS
	T1_4	9	9	0	1.000	**0.004**	**	6	3	0.667	0.508	NS
LFH_19	M2_2	10	10	0	1.000	**0.002**	**	7	3	0.700	0.344	NS
	T1_4	10	10	0	1.000	**0.002**	**	6	4	0.400	0.754	NS
LFH_20	M3_19	10	10	0	1.000	**0.002**	**	-	-	-	-	
	M2_2	10	-	-	-	-		7	3	0.300	0.344	NS
	T2_6	10	10	0	1.000	**0.002**	**	-	-	-	-	
	T2_7	8	-	-	-	-		4	4	0.500	1.000	NS
	T1_4	10	9	1	0.900	0.021	*	-	-	-	-	
LFH_21	M3_10	10	-	-	-	-		8	2	0.200	0.109	NS
	M3_19	10	-	-	-	-		7	3	0.700	0.344	NS
	M2_2	9	9	0	1.000	**0.004**	**	5	4	0.444	1.000	NS
	M2_19	8	8	0	1.000	**0.008**	**	-	-	-	-	
	T2_7	10	10	0	1.000	**0.002**	**	-	-	-	-	
	T4_5	10	10	0	1.000	**0.002**	**	6	4	0.600	0.754	NS
LFH_24	M2_2	10	-	-	-	-		5	5	0.500	1.000	NS
	M2_3	10	10	0	1.000	**0.002**	**	-	-	-	-	
	T2_7	10	-	-	-	-		6	4	0.400	0.754	NS
	T4_5	10	10	0	1.000	**0.002**	**	-	-	-	-	
	T1_4	10	10	0	1.000	**0.002**	**	-	-	-	-	
LFH_25	M3_19	10	10	0	1.000	**0.002**	**	8	2	0.800	0.109	NS
	T2_6	9	9	0	1.000	**0.004**	**	-	-	-	-	
	T2_7	10	10	0	1.000	**0.002**	**	6	4	0.600	0.754	NS
	T1_4	10	-	-	-	-		6	4	0.400	0.754	NS
LFH_26	M3_19	10	10	0	1.000	**0.002**	**	-	-	-	-	
	T4_5	10	10	0	1.000	**0.002**	**	-	-	-	-	
LFH_28	M2_2	10	10	0	1.000	**0.002**	**	5	5	0.500	1.000	NS
	T4_5	10	10	0	1.000	**0.002**	**	6	4	0.400	0.754	NS
	T1_4	10	-	-	-	-		7	3	0.700	0.344	NS
LFH_29	M3_10	10	-	-	-	-		6	4	0.600	0.754	NS
	M3_19	10	-	-	-	-		6	4	0.600	0.754	NS
	M2_2	10	-	-	-	-		7	3	0.300	0.344	NS
	T2_7	10	10	0	1.000	**0.002**	**	5	5	0.500	1.000	NS
	T4_5	10	10	0	1.000	**0.002**	**	-	-	-	-	
	T1_4	10	-	-	-	-		6	4	0.600	0.754	NS
LFH_30	M3_10	10	-	-	-	-		8	2	0.200	0.109	NS
	M3_19	10	10	0	1.000	**0.002**	**	-	-	-	-	
	M2_2	10	-	-	-	-		6	4	0.400	0.754	NS
	T2_6	9	9	0	1.000	**0.004**	**	-	-	-	-	
	T2_7	10	-	-	-	-		7	3	0.300	0.344	NS
LFH_31	M3_10	10	-	-	-	-		7	3	0.700	0.344	NS
	M3_19	10	-	-	-	-		5	5	0.500	1.000	NS
	M2_2	9	9	0	1.000	**0.004**	**	5	4	0.556	1.000	NS
	T2_6	10	10	0	1.000	**0.002**	**	5	5	0.500	1.000	NS
	T2_7	10	10	0	1.000	**0.002**	**	-	-	-	-	
	T4_5	10	10	0	1.000	**0.002**	**	-	-	-	-	
	T1_4	10	-	-	-	-		6	4	0.600	0.754	NS
LFH_33	M3_10	9	-	-	-	-		5	4	0.444	1.000	NS
	M2_2	10	9	1	0.900	0.021	*	6	4	0.600	0.754	NS
	T2_6	10	10	0	1.000	**0.002**	**	7	3	0.300	0.344	NS
	T2_7	9	9	0	1.000	**0.004**	**	5	4	0.556	1.000	NS
	T4_5	9	9	0	1.000	**0.004**	**	7	2	0.333	0.180	NS
	T1_4	9	-	-	-	-		6	3	0.667	0.508	NS
LFH_34	M2_2	7	7	0	1.000	0.016	*	5	2	0.286	0.453	NS
	T2_6	10	10	0	1.000	**0.002**	**	-	-	-	-	
	T2_7	10	9	1	0.900	0.021	*	6	4	0.400	0.754	NS
	T4_5	10	10	0	1.000	**0.002**	**	6	4	0.600	0.754	NS
	T1_4	10	-	-	-	-		7	3	0.300	0.344	NS
**Body louse**												
LFB_01	M3_10	5	5	0	1.000	0.063	NS	-	-	-	-	
	M3_19	5	-	-	-	-		3	2	0.400	1.000	NS
	M2_13	5	4	1	0.800	0.375	NS	-	-	-	-	
	T4_5	6	6	0	1.000	0.031	*	-	-	-	-	
LFB_02	M2_3	9	-	-	-	-		6	3	0.667	0.508	NS
	T4_5	10	10	0	1.000	**0.002**	**	-	-	-	-	
LFB_04	M3_19	7	-	-	-	-		4	3	0.571	1.000	NS
	M2_19	7	-	-	-	-		6	1	0.143	0.125	NS
	M2_3	7	7	0	1.000	0.016	*	-	-	-	-	
	T4_5	8	-	-	-	-		5	3	0.375	0.727	NS
LFB_06	M3_10	10	-	-	-	-		6	4	0.600	0.754	NS
	M3_19	10	10	0	1.000	**0.002**	**	-	-	-	-	
	M2_19	7	7	0	1.000	0.016	*	-	-	-	-	
	M2_3	10	-	-	-	-		7	3	0.300	0.344	NS
	T2_6	10	7	3	0.700	0.344	NS	-	-	-	-	
	T4_5	18	14	4	0.778	0.031	*	-	-	-	-	
LFB_07	M3_19	8	-	-	-	-		5	3	0.625	0.727	NS
	M2_3	10	10	0	1.000	**0.002**	**	-	-	-	-	
	T4_5	10	10	0	1.000	**0.002**	**	6	4	0.600	0.754	NS
LFB_08	M3_10	10	-	-	-	-		8	2	0.200	0.109	NS
	M2_2	9	-	-	-	-		6	3	0.667	0.508	NS
	T2_6	10	-	-	-	-		6	4	0.400	0.754	NS
	T4_5	10	-	-	-	-		7	3	0.700	0.344	NS
LFB_09	M3_10	10	10	0	1.000	**0.002**	**	6	4	0.400	0.754	NS
	T4_5	10	10	0	1.000	**0.002**	**	-	-	-	-	
	T2_6	10	5	5	0.500	1.000	NS	-	-	-	-	
	M2_19	7	-	-	-	-		4	3	0.571	1.000	NS
LFB_10	M3_10	10	10	0	1.000	**0.002**	**	6	4	0.400	0.754	NS
	M3_19	6	6	0	1.000	0.031	*	-	-	-	-	
	T4_5	10	10	0	1.000	**0.002**	**	-	-	-	-	
LFB_14	M3_10	10	-	-	-	-		5	5	0.500	1.000	NS
	M3_19	9	-	-	-	-		6	3	0.333	0.508	NS
	M2_2	10	10	0	1.000	**0.002**	**	-	-	-	-	
	M2_3	10	10	0	1.000	**0.002**	**	-	-	-	-	
	T4_5	10	10	0	1.000	**0.002**	**	5	5	0.500	1.000	NS
LFB_15	M3_10	9	-	-	-	-		5	4	0.444	1.000	NS
	M2_13	16	16	0	1.000	**<0.001**	**	-	-	-	-	
	M2_3	21	20	1	0.952	**<0.001**	**	13	8	0.381	0.383	NS
	T4_5	21	19	2	0.905	**<0.001**	**	12	9	0.429	1.000	NS
LFB_16	M3_10	17	17	0	1.000	**<0.001**	**	9	8	0.471	1.000	NS
	M2_2	16	16	0	1.000	**<0.001**	**	-	-	-	-	
	M3_19	10	10	0	1.000	**0.002**	**	6	4	0.400	0.754	NS
	M2_19	10	-	-	-	-		7	3	0.700	0.344	NS
	M2_3	10	-	-	-	-		6	4	0.400	0.754	NS
LFB_19	M3_19	10	-	-	-	-		5	5	0.500	1.000	NS
	M2_13	10	-	-	-	-		5	5	0.500	1.000	NS
LFB_24	M3_19	-	-	-	-	-		5	3	0.625	0.727	NS
	T2_6	10	10	0	1.000	**0.002**	**	-	-	-	-	
	T4_5	10	10	0	1.000	**0.002**	**	-	-	-	-	
